# KLF10 as a Tumor Suppressor Gene and Its TGF-β Signaling

**DOI:** 10.3390/cancers10060161

**Published:** 2018-05-25

**Authors:** Azra Memon, Woon Kyu Lee

**Affiliations:** Laboratory of Developmental Genetics, Department of Biomedical Sciences, School of Medicine, Inha University, Incheon 22212, Korea; azrabiochem@yahoo.com

**Keywords:** KLF10, TGF-β, disease, cancer, tumor suppressor

## Abstract

Krüppel-like factor 10 (KLF10), originally named TGF-β (Transforming growth factor beta) inducible early gene 1 (TIEG1), is a DNA-binding transcriptional regulator containing a triple C2H2 zinc finger domain. By binding to Sp1 (specificity protein 1) sites on the DNA and interactions with other regulatory transcription factors, KLF10 encourages and suppresses the expression of multiple genes in many cell types. Many studies have investigated its signaling cascade, but other than the TGF-β/Smad signaling pathway, these are still not clear. KLF10 plays a role in proliferation, differentiation as well as apoptosis, just like other members of the SP (specificity proteins)/KLF (Krüppel-like Factors). Recently, several studies reported that KLF10 KO (Knock out) is associated with defects in cell and organs such as osteopenia, abnormal tendon or cardiac hypertrophy. Since KLF10 was first discovered, several studies have defined its role in cancer as a tumor suppressor. KLF10 demonstrate anti-proliferative effects and induce apoptosis in various carcinoma cells including pancreatic cancer, leukemia, and osteoporosis. Collectively, these data indicate that KLF10 plays a significant role in various biological processes and diseases, but its role in cancer is still unclear. Therefore, this review was conducted to describe and discuss the role and function of KLF10 in diseases, including cancer, with a special emphasis on its signaling with TGF-β.

## 1. SP/KLF Family

Transcription factors SP (specificity proteins) and KLF (Krüppel-like Factors) contain three Krüppel-like zinc fingers collectively known as the SP/KLF family. While members of this family also have a unique amino-terminal (N-terminal) end that acts as the functional domain and that allows it to bind specifically to a certain partner, the N-terminal domains of the various KLFs vary, and these different domains mediate different molecular functions [1]. In the early 1980s, SP1 was first identified as a protein capable of binding GC and GT rich regions or the 5′-CACCC-3′ DNA sequence in the SV40 promoter, indicating that it could serve as a transcriptional regulator [2]. To date, a total of 27 identified members in mammals (human) have been characterized, from which nine members of the SP subfamily (SP1–SP9) and 18 members of the KLF family (KLF1–KLF18) were included (Table 1) [3,4,5]. They were categorized as members of SP/KLF because of their resemblance in the zinc finger motif located toward the C-terminus [6]. Buttonhead (Btd) box domain CXCPXC, which differentiate SP from KLFs is only present in the SP family at just 5′ of the KLF DNA binding domain [7], whereas N-terminal regions of KLF/SP transcription factors are extremely flexible and consist of different combinations of transactivation/repression domains.

SP/KLF factors have been found to be engaged in fundamental cell processes such as growth, differentiation, apoptosis, and angiogenesis; therefore, the family is involved in numerous aspects of tumorigenesis, while other diseases behave as either activators or repressors (Table 1). Mammalian KLFs have been divided into 3 groups based on the shared domain. Most of the members of this family are widely expressed, but some have restricted tissue expression. Formerly these KLFs were named as they were identified, KLF10 and KLF11 were originally identified as early genes induced by transforming growth factor β and were named TGF-β inducible early gene 1 (TIEG1) and TGF-β inducible early gene 2 (TIEG2), respectively [8,9]. Conversely, KLF1 expressed in specific tissues erythroid and therefore referred to as EKLF [6], KLF2 is found in the lung and therefore referred to as LKLF [10], KLF3 is widely expressed and therefore called basic BKLF [11], KLF4 is initially found gut enriched KLF and called GKLF [12,13], KLF5 is found in the intestines and therefore known as IKLF [14], and KLF15 is initially found in the kidney so it is referred to as KKLF. KLF9 was identified as a basal transcription element binding (BTEB) protein [13], with KLF5 and KLF13 as homologs (BTEB2 and BTEB3, respectively) [15], while KLF17 was first discovered as a germ cell-specific gene encoding a zinc finger protein 393 (Zfp393) [16]. KLF18 genes/pseudogenes are found in most placental mammals (for example, human, mouse) [3].

**Figure 1:**
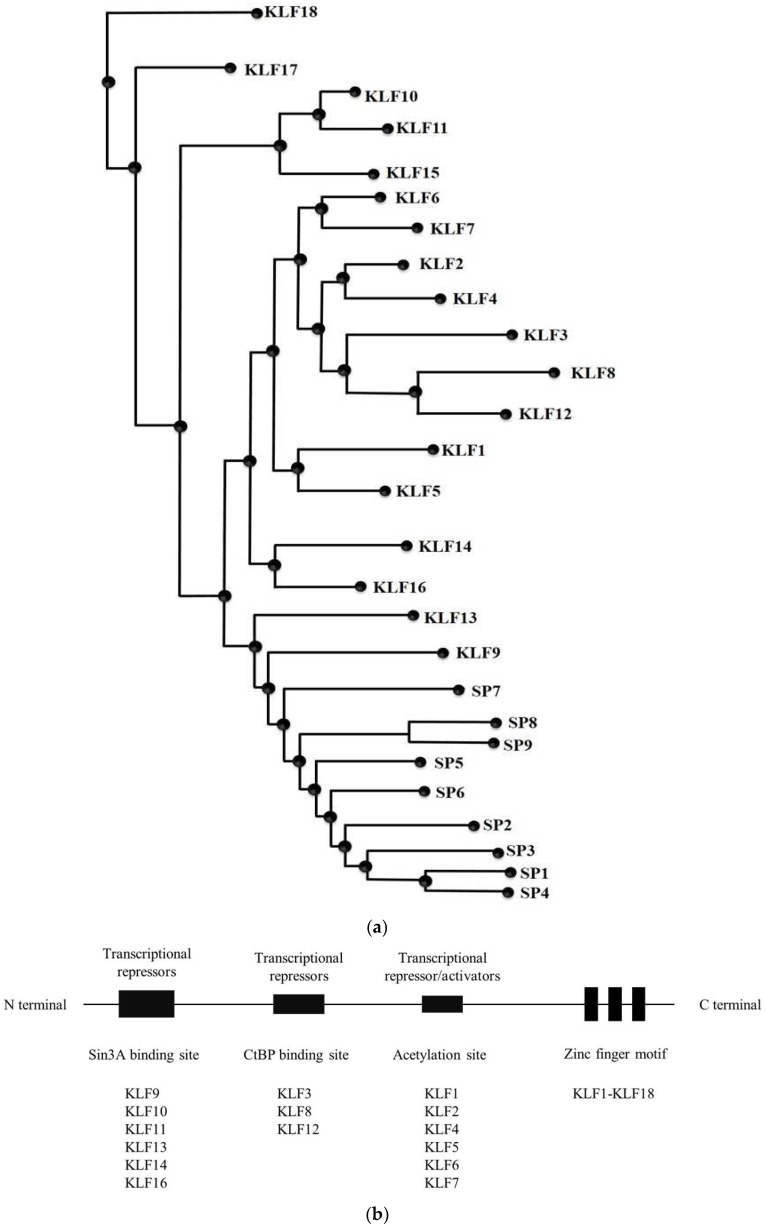

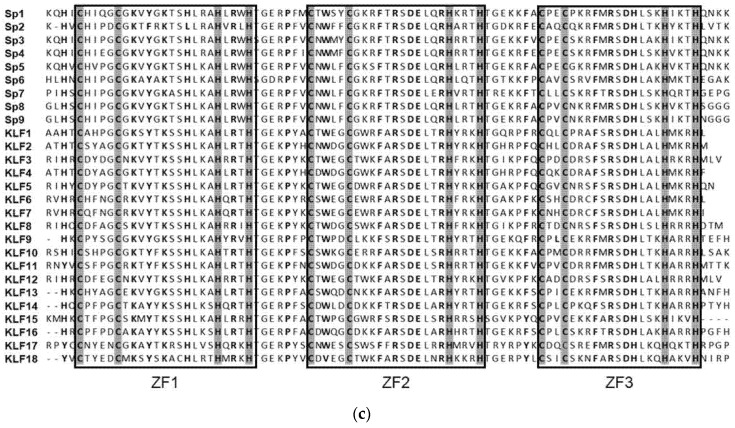
Phylogenic tree, common structure and zinc finger motif in SP (specificity proteins)/KLF (Krüppel-like Factors) family. (**a**) Phylogenic analysis based on human protein sequence from NCBI. (**b**) Structure of KLF family, KLFs isoforms can be divided into 3 groups based on the interaction of their N-terminal site with other proteins for transcriptional co-activators and co-repressors. Group 1 includes KLFs that contain the CtBP binding site. Group 2 includes KLFs that contain the Sin3A interaction domain. Group 3 includes KLFs that interact with acetyl-transferases. KLF15, KLF17, and KLF18 are not included in any of these groups because little is known about their protein interaction motifs and (**c**) multiple alignments of zinc finger domain of all family members of human Sp/KLF factors. Each protein contains three zinc fingers motifs at the C-terminus, two conserved cysteine residues and two conserved histidine residues for zinc binding are highlighted.

The key feature of SP/KLF proteins is the presence of a DNA-binding domain consisting of three Krüppel-like zinc fingers classified into different subfamilies based on the presence of conserved motifs within their N-terminal domains outside the zinc finger domain (Figure 1). KLF members are divided into distinct groups according to functional similarities revealed upon structural analysis (reviewed in ref [17]. Most KLF proteins were randomly named by their discoverers, which led to the development of a new nomenclature by the HGNC (Human Gene Nomenclature Committee) that serially designates each KLF protein by number [18].

Several groups have described various approaches to identify all of the members of the SP/KLF family in human and mouse genomes, and the effects of these proteins in more detail [4,19,20,21,22,23,24,25]. As shown in Figure 1, the protein-binding domains within the protein structures of KLF Group 1 (KLF 3, 8, and 12) serve as transcriptional repressors through their interaction with the C-terminal binding protein (CtBP). Group 2 (KLF 1, 2, 4, 5, 6, and 7) function mostly as transcriptional activators and bind to histone acetyltransferases (HATs) and Group 3 (KLF 9, 10, 11, 13, 14, and 16) have repressor activity through the presence of a Sin3A-binding site. KLF 15, 17 and 18 based s contain no defined protein interaction motifs. Eighteen mammalian KLFs have been recognized to date, and these have received increased attention because of their basic, diverse biological processes and their contributions to human diseases (Table 1). Here, we reviewed KLF10 to understand its role and function in diseases, including cancer as a tumor suppressor, as well as its interaction with TGF-β signaling.

## 2. KLF10 Induced Mechanism of Gene Activation

Transforming growth factor (TGFβ) beta-like group proteins consist of a large family of related growth factors comprised of at least 30 members in mammals. TGFβ has three isoform TGFβ1, 2, 3 which function as primary mediators of TGFβ signaling. These three ligands are structurally related to activins, nodals and some growth and differentiation factors (GDFs), and the bone morphogenetic proteins (BMP) [26]. TGFβ superfamily members regulate fundamental cell processes such as proliferation, differentiation, death, cytoskeletal organization, adhesion, and migration. Smad proteins are the major intracellular mediators of TGFβ signaling pathway to control transcription of their target genes [27]. TGF-β1 activates SMAD-dependent and -independent pathways to exhibit its biological activities [28]. Members of the SMAD are classified into three groups, R-SMADs, the receptor-regulated SMADs which include SMAD1, SMAD2, SMAD3, SMAD5, and SMAD8, Co-SMAD (common SMAD) which have only one member SMAD4, I-SMADs (inhibitory SMADs) including SMAD6 and SMAD7. It is well-known that TGF-β is stimulated by activating downstream mediators SMAD2 and SMAD3, and is negatively regulated by an inhibitory SMAD7 [29].

In addition, several KLFs are linked to TGFβ signaling, TGFβ induces the expression of early response transcription factors such as the Sp1/KLF like zinc-finger proteins KLF10 and KLF11 [9,30]. KLF10, which work as effector proteins in TGFβ mediated cell growth control and differentiation were originally defined as TGF-β inducible early gene 1 (TIEG1) in human osteoblast (OB) cells [10]. The KLF10 N-terminal protein segments also contain three unique repression domains, R1, R2, and R3 [10,11,12]. The N terminal region of the Sp1-like transcriptional factor family is variable, therefore, these three transcriptional repressor domains are a critical feature of the KLF10 and KLF11 subfamily. GC-rich sequences of KLF10 play important roles in the regulation of a large number of genes essential for various cellular functions, including cell proliferation, differentiation, and apoptosis [13]. KLF10 is involved in several different types of gene expression in various cell types and serves as a target gene for many signaling pathways. This gene is known to be induced by estrogen, TGF-βs, bone morphogenetic protein (BMP), nerve growth factor and epidermal growth factor (EGF) depending on cellular and environmental circumstances. One of its potential roles as a transcription factor is that it mimics the anti-proliferative effect of TGF-β and induces apoptosis [31]. KLF10 and TGF-β induce apoptosis by the formation of ROS (reactive oxygen species) and a loss of the mitochondrial membrane potential [32]. KLF10 facilitates TGF-β signaling after phosphorylation of the carboxy-terminal serine residue of the internal modulator Smad2/Smad3 protein via TGF-β receptor type I, and interaction with Smad4, the nuclear localized Smad compound, induces expression of KLF10 in order to bind with promoters of Smad2, Smad7, and TGF-β1. KLF10 overexpression increases endogenous TGF-β regulated genes p21 and PAI-1 [33]. Additionally, KLF10 is regulated by other members of the TGF-β superfamily, such as BMPs, activins and GDNF (glial cell derived neurotrophic factor), which suggests that KLF10 might act in diverse signaling pathways at the transcriptional level.

## 3. KLF10 Role in Various Diseases

KLF10 has the potential for use as a marker for various diseases including diabetes, cardiac hypertrophy, and osteoporosis (Table 2).

### 3.1. Diabetes

High blood and tissue concentrations of glucose play a significant role in the development of vascular complications in diabetes mellitus (DM) patients. Loss of KLF10 in liver tissue suppresses glycolytic proteins and encourages gluconeogenic and lipogenic proteins [43]. KLF10 is a circadian-clock-controlled transcription factor that can suppress lipogenic genes involved in glucose and lipid metabolism in the liver [44] that also affects hepatic gluconeogenesis, which contributes to T2DM [36]. However, no apparent pathological defects of pancreatic function were observed under basal conditions in knockout mouse models of KLF10 [45,46]. Using real-time PCR analysis, Zitman-Gal et al. found that the expression of KLF10 was also upregulated in a diabetic-like environment, whereas the addition of calcitriol significantly down-regulated KLF10 mRNA expression [47].

### 3.2. Bone Disease

TGF-β, which plays a major role in osteoblast (OBL) and osteoclast (OCL) functions, is present in large amounts in the skeleton [48]. KLF10 is one of the key transcriptional regulators in osteogenesis regulated through the TGF-β signaling pathway [49]. KLF10 knock-out (KO) mice have slow osteoblast production of RANKL (receptor activator of nuclear factor kappa-B ligand) and high levels of osteoprotegerin (OPG), which delays OCL differentiation, leading to a reduced bone turnover and a loss of bone (osteopenia) [34,35,46]. KLF10 also shows osteoclast precursors that differentiate slowly, as well as increased AKT (a serine/threonine-specific protein kinase) and MAPK/ERK (mitogen activated protein kinases/extracellular signal regulated kinases) signaling pathway activation, which is consistent with the roles of these kinases in promoting osteoclast survival. Higher RANKL concentrations can restore this defect, suggesting that KLF10 plays a role in osteogenesis through RANKL signaling [50].

### 3.3. Heart Hypertrophy

Microarray analysis of heart tissue from the left ventricles of KLF10 KO male, but not female, mice aged 16 months showed a 14-fold increase, increased fibrosis, and increased wall thickness relative to wild-type animals [37]. Another study reported that KLF10 is downregulated in Angiotensin II (Ang II) induced hypertrophy through repression of expression of the cardiac transcription factor GATA4 and mRNA levels of hypertrophy-related genes atrial natriuretic factor (ANF) and brain natriuretic peptide (BNP) [51]. TGF-β1 and KLF10 control T regulatory cell differentiation and suppressor function and act as regulators of CD4^+^CD25^−^ T cell activation. KLF10 in response to TGF-β1 can transactivate both TGF-β1 in CD4+CD25^−^ T cells and Foxp3 promoters, which is associated with a positive response of KLF10 expressed highly in T regulatory cells [38]. KLF10 plays a critical role in the regulation of atherosclerotic lesion formation in mice by targeting TGF-β1 to regulate CD4+CD25^−^ T cells and T regs. KLF10 also complexed with pituitary tumor-transforming gene-1 (Pttg1), which is one of its target genes and plays an important role in cardiac hypertrophy [37,52].

### 3.4. Other Diseases

KLF10 are able to physically associate withthe FOXP3 gene to induce transcription and can either positively or negatively regulate FOXP3 through its differential association with p300/CBP-associated factor (PCAF) or the histone deacetylase binding protein Sin3, respectively. Inactivation of immune genes, such as FOXP3, may cause human diseases such as colitis [53]. In the absence of KLF10, colonic macrophages express lower levels of TGFβRII and reduced Smad2 phosphorylation, resulting in TGF-β1 stimulation that contributes to colitis [41,54]. Moreover, transcriptomic analysis of peritoneal cells in a mouse model of sepsis caused by infection with a non-pathogenic strain of *Escherichia coli* revealed that KLF10 was down-regulated at 2 h [45], while KLF10 induces TGF-β1 expression, which is an anti-inflammatory cytokine and regulates T cell activation [29]. KLF10 expression was significantly increased in diet-induced nonalcoholic steatohepatitis (NASH) and collagen-producing activated hepatic stellate cells. This up-regulation of KLF10 increased TGF-β signaling genes and suppressed ChREBP expression [40]. Furthermore, KLF10 plays a role in hyperglycemia [55], human chronic obstructive pulmonary diseases and liver cirrhosis [56], tendon repair [57], and hypoxia [58]. However, the actual functional role and mechanism of KLF10 in various pathophysiological conditions are still uncertain.

### 3.5. Phenotype in KLF10 Deficient Models

KLF10 deficient animals are widely used to determine its role in different cellular process and diseases. The KO phenotype of KLF10 shows a normal lifespan in mice, but with some defects such as in the microarchitecture and mechanical properties of tendons. The tail tendons of KLF10 KO mice were significantly less stiff than the wild-type controls at 3 months of age, while no difference existed at 1 or 15 months, indicating that there are age-dependent changes in the mechanical properties of the tendon in KLF10 KO mice [59]. KLF10 display an important role in skeletal development and homeostasis; significantly weaker bones and reduced amounts of cortical and trabecular bone were noticed in the absence of KLF10 [60]. Additionally, transmission electron microscopy revealed that osteocytes display defects in their morphology, density and surrounding bone matrix [61]. Moreover, type-I collagen, which is the most abundant collagen found in tendons, was significantly decreased in KLF10 KO tendons [62].

KO of KLF10 shows gender-specific osteopenic phenotypes in females characterized by a decrease in the total number of functional/mature OBLs indicating a potential role of KLF10 in mediating estrogen signaling (as well as TGFβ) in the skeleton [63,64,65]. Moreover, KLF10 null mice develop age-related hypertrophy [66]. Loss of KLF10 can delay wound healing and increase Smad 7 in wounds, which causes weakened wound contraction, granulation tissue formation, collagen synthesis, and re-epithelialization [32,33]. Its loss has reduced endothelial progenitor cells (EPCs) function and TGF-β1 responsiveness because of impaired blood flow recovery after hindlimb ischemia in systemic KLF10−/− mice [67]. KLF10 lacking bone marrow display both basal defects in their ability to migrate and incorporate into the vessel wall and complex paracrine defects in their ability to facilitate endothelial cell (EC) growth and migration [68].

## 4. Role of KLF10 as a Tumor Suppressor in Various Cancers

**Figure 2:**
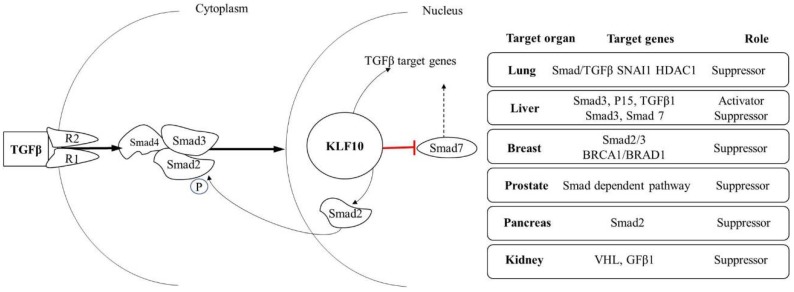
TGF-β signaling and its target disease mediated by KLF10 gene. In response to TGFβ ligand binding, KLF10 gene expression is induced in a Smad dependent manner and play a role as a tumor suppressor in many cancers. TGFβ signaling occurs through TGFβ receptors (TβRI and TβRII). Binding of TGFβ ligand to TβRII facilitates phosphorylation of TβRI, which in turn phosphorylates the SMAD2 and SMAD3 proteins. After phosphorylationSmad2/Smad3 form complex with Smad4, and translocate to the nucleus to induce expression of KLF10. Subsequently, KLF10 bind to the promoters of Smad2, Smad7, and TGF-beta1, whereas the expression of the inhibitory Smad7 is blocked to disrupt the negative feedback loop. Importantly, KLF10 serves as a positive feedback loop for regulating TGFβ signaling by inducing the expression of SMAD2 and inhibiting the expression of the inhibitory SMAD7 gene in many cancers.

KLF10 plays a vital role in many biological processes and diseases, including tumorigenesis (Table 3). Many studies have shown that KLF10 acts as a tumor suppressor through TGF-β signaling by playing an important role in inhibition of cell proliferation and induction of apoptosis (Figure 2) [30,69]. KLF10 is an effective repressor of cancer cell proliferation, overexpression of KLF10 reduced cell proliferation in many cancer types while in the absence of KLF10 cell proliferation increased, which decreases Smad dependent transcription, and importantly, Smad2/3 in association with a prolonged increase in Smad7 expression. Moreover, KLF10 is a valuable tool to enhance the death of p53 deficient cancer in association with low dose chemotherapy [70].

### 4.1. Liver Cancer

Loss of TGF-β sensitivity has been assumed to be an event in HCC (hepatocellular carcinoma) development [84,85]. Activation of KLF10 gene can promote growth inhibition and apoptosis of TGF-β-susceptible human HCC cells, as well as to inhibit stathmin promoter activity, suggesting stathmin is a direct target of KLF10 [79]. Furthermore, KLF10 was found to repress glutathione transferase P (GST-P) promoter activity, which is an excellent tumor marker in hepatocarcinogenesis, by binding to GST-P silencer 2 [86]. Lack of KLF10 blocked cellular proliferation of hepatocytes by encouraging TGF-β/Smad pathway during liver tumorigenesis [80]. Koczulla et al. reported that KLF10 is highly expressed in liver cirrhosis [56]; however, there is inadequate information regarding KLF10, and the actual functions of KLF10 in liver tumorigenesis are controversial.

### 4.2. Pancreatic Cancer

KLF10 expression is inversely associated with pancreatic cancer and therefore, KLF10 can be used as a predictive indicator for pancreatic cancer stages [87]. KLF10 is expressed in both acinar and ductular epithelial cell populations and plays a significant role in the pancreatic β-cells. Loss of this gene increases p21^Cip1^ in islet cells, which are associated with impaired glucose tolerance and impaired insulin secretion [30,88]. KLF10 overexpression in the TGF-β sensitive pancreatic human cell line is sufficient to induce apoptosis [89]. Furthermore, its overexpression-induced cell cycle arrest at the G1- to S-phase transition to inhibit human pancreatic cancer SW1990 cell proliferation [77]. KLF10 is located on chromosome 8q22, where mutations are quite frequent in pancreatic cancers [90]; however, mutational screening of a panel of the twenty-two human pancreatic cell lines showed no alteration in KLF10 expression [78].

### 4.3. Lung Cancer

KLF10 occupied GC-rich sequences in the promoter region of EMT (epithelial mesenchymal transition) promoting transcription factor SLUG/SNAI2 (snail family zinc finger 2) and acted as the main factor of the TGF-β1 related EMT program [91]. Mishra et al. reported that KLF10 suppresses TGF-β induced EMT by binding and repressing the SNAI2 promoter as a direct KLF10 target gene through HDAC1 in lung and pancreatic cancer models [82]. Furthermore, in vitro and in vivo studies showed decreased tumor growth and increased chemo-sensitivity to gemcitabine through G0/G1 cell cycle arrest after Cul4A (cullin 4A) knockdown, which is an important regulator of proliferation and cell cycle progression in lung cancer. In addition, Cul4A knockdown increases the expression of KLF10, cyclin-dependent kinase inhibitor p21/WAF1, and TGF-β1, which play a role as tumor suppressors [92].

### 4.4. Breast Cancer

KLF10 and its target genes play important roles in breast cancer. KLF10 has been indicated as a marker for breast cancer, and different stages of breast tumor tissues from different populations show decreased mRNA levels of KLF10, Smad2, and BRAD1 (breast cancer 1 associated RING domain 1), while Smad7 is inversely correlated with KLF10 [73]. KLF10 is an anti-metastasis gene that significantly prevents breast cancer cell invasion, suppresses mammary tumorigenesis and decreases lung metastasis by inhibition of EGFR (epidermal growth factor receptor) gene transcription through the EGFR signaling pathway [93]. Furthermore, Hsu et al. reported a significant E2/Klf10/BI-1/cytoplasmic calcium pathway in which E2 (estrogen) induces apoptosis through increased expression of KLF10, which decreases BI-1 (Bax inhibitor-1) transcription and finally increases the concentration of Ca^2+^(cytoplasmic calcium) [94]. The above studies strongly support KLF10 as a tumor suppressor protein that plays an inhibitory role in the proliferation of breast cancer.

### 4.5. Colon Cancer

KLF10 is believed to be a tumor suppressor gene in many cancers (Table 2), including human colorectal cancer. Additionally, it is one of the members of the signal transduction of the peroxisome proliferator-activated receptor gamma (PPARγ) pathway in human colorectal cancer cells. Human colorectal cancer cells express abundant PPARγ, but its inhibitory function is very low, signifying a defect in the PPARγ pathway [95]. Treatment of colon cancer cells with 15-hydroxy-eicosatetraenoic acid (15S-HETE), an endogenous ligand for PPARγ, arrests the growth of colon cancer cells via a PPARγ dependent pathway involving increased expression of KLF10 and decreased Bcl-2 (B cell lymphoma 2) expressions [72].

### 4.6. Human Prostate Cancer

Instigation of the TGF-β1 pathway triggers doxazosin-based apoptotic effect in prostate cancer cells. Doxazosin is an α1-selective alpha blocker used to treat high blood pressure and urinary retention related to benign prostatic hyperplasia (BPH). In vitro analysis has shown that treatment of PC-3 prostate cancer cells with doxazosin resulted in a marked induction in KLF10 and Smad4 mRNA levels, as well as a transient decrease in Smad7 mRNA expression. Smad4 is an important regulator of TGF-β1 signaling and apoptosis in a variety of cell lines [71,96,97].

### 4.7. Metastatic Brain Tumors

Metastasis is a procedure in which cancerous cells spread from the initial or primary tumor, enter the bloodstream or lymphatic system, and spreads to different or secondary sites within the body in numerous malignant neoplasms, including breast, lung, and ovarian cancer. Gene expression profiling using a 17k-expression array of metastatic brain tumors from primary lung adenocarcinoma revealed that KLF10, which is a gene involved in apoptosis, was expressed at insufficient or abnormally low levels in metastatic brain tumors, suggesting KLF10 acts as a tumor-repressor gene [75].

### 4.8. Renal Cancer

KLF10 facilitates up-regulation of TGF-β1 in VHL (von Hippel-lindau) deficient tumors. In a Luc-reporter assay, a potential KLF10 binding site was identified in the TGF-β1 promoter upstream from the transcription initiation site, which is also recognized as a Sp1-binding site. KLF10 was suppressed in the renal cancer cell line by wild-VHL and not from mutant VHL cells, suggesting that it may also serve as a VHL target. Stimulation of the TGF-β1 promoter by KLF10 in HAEo(−) and 293 cells suggests that KLF10 is a VHL target that regulates the TGF-β1 promoter in renal cell carcinoma [76].

### 4.9. Other Cancers

Smad signaling is present in certain different human lymphoma cells. However, in lymphoma cancer, the contribution of Smads to TGF-β induced apoptosis is supported by the increased expression of KLF10, which is a Smad-responsive gene [74,98]. KLF10 has been recognized as a direct downstream target of miR-410 in multiple myelomas (MM) cells and facilitates the effects of miR-410 in MM, resulting in PTEN (phosphatase and tension homolog)/AKT activation [42,99]. KLF10 is a circadian transcriptional regulator that links the molecular clock to energy metabolism [44]. In ovarian cancer, transcription factor KLF10 activated by circadian rhythm gene expression serves as a risk factor for epithelial ovarian cancer, histopathologic subtype, and invasiveness [81]. The zinc finger transcription factor KLF10 regulates myeloid-specific activation of the leukocyte integrin CD11d promoter [100]. Co-transfection and electrophoretic mobility shift have revealed that KLF10 competes with these SP proteins for binding to overlapping sites in the CD11d promoter. Stimulation of CD11d expression resulting in differentiation of myeloid cells is mediated through increased binding of KLF10 to the CD11d promoter [101].

## 5. Concluding Remarks

KLF10 plays an active role in the etiology and development of many mammalian diseases and appears to have unique tissue-specific roles that were identified primarily using in vivo experiments, including gene knockout models. KLF10 is believed to play a crucial role in inhibition of cancer cell proliferation and promotion of apoptosis, which strongly indicate its role as a tumor suppressor. However, there is limited information regarding the mechanism of its role in cancer. The majority of the KLF10 role and gene targeting is mediated through TGF-β signaling. Taken together, the results of the studies discussed indicate that KLF10 can have profound effects as a tumor suppressor in many cancers via TGF-β/Smad dependent and independent pathways. Further understanding of its function and target genes are needed to provide additional insights into the mechanisms of action of KLF10 to understand its role in diseases, including cancer.

## Figures and Tables

**Table 1:** SP (specificity proteins)/KLF (Krüppel-like Factors) family of transcription factors and their expression.

Name	Previous Name	Transcription Activity	Expression Pattern	Disease	TGFβ Signaling
**Sp1**	TFSP1	Activator	Ubiquitous	Alzheimer’s disease (AD)	Co-activator of Smad-dependent transduction pathway in AD
**Sp2**	KIAA0048	Activator/Repressor	Unknown	Unknown	Unknown
**Sp3**	SPR-2	Activator/Repressor	Ubiquitous	Pathogenesis of keratoconus	SP1/Sp3 activities control TGFβRII gene
**Sp4**	SPR-1, HF1B	Activator/Repressor	Brain enriched	Unknown	Unknown
**Sp5**		Unknown	Ubiquitous	Unknown	Unknown
**Sp6**		Activator	Ubiquitous	Unknown	Unknown
**Sp7**	OSX	Unknown	Osteoblastic cells	Bone cell differentiation	Unknown
**Sp8**	BTD	Unknown	Neurogenic regions	Neural tube formation	Unknown
**Sp9**	ZNF990	Unknown	Unknown	Unknown	Embryonic limb morphogenesis
**KLF1**	E-KLF	Activator	Erythropoietic tissues (fetal liver and adult bone marrow)	Anemia β-thalassemia	Unknown
**KLF2**	L-KLF	Activator	Ubiquitous	Glomerular disease, atherosclerosis, vascular inflammation, cancers (leukemia, breast, colon, intestine, prostate)	Inhibits TGF-β signaling in atherosclerosis
**KLF3**	BKLF, TEF-2	Activator/Repressor	Ubiquitous	Cancer (leukemia, cervix)	Unknown
**KLF4**	G-KLF, EZF	Activator/Repressor	Ubiquitous	Glomerular disease, IBD, acute kidney injury, liver fibrosis, heart failure, axon regeneration, different types of cancers (bladder, brain, breast, cervix, colon, intestine, esophagus, head and neck, liver, leukemia, lung, lymphoma, prostate, skin stomach, melanoma, pancreas)	Cell proliferation and differentiation, important target in macrophages
**KLF5**	I-KLF, C-KLF, BTEB2	Activator/Repressor	Gut and epithelial tissue, Placenta	IBD, kidney fibrosis, different types of cancers (leukemia, breast, colon, intestine, esophagus, head and neck, gastrointestinal stromal tumor, lung) pancreas, melanoma, prostate, stomach)	Proliferation, TGFβ induced growth arrest
**KLF6**	BCD1, COPEB, CPBP, GBF, PAC1, ST12, Zf9	Activator	Ubiquitous	Cardiac fibrosis, kidney fibrosis, different types of cancers (leukemia, bone, breast, brain, colon, intestine, head and neck, liver, lung, ovary, pancreas, pituitary, prostate, stomach)	Cell proliferation in skeletal myoblasts
**KLF7**	U-KLF	Activator	Ubiquitous	Type 2 diabetes	Satellite cell quiescence
**KLF8**	BKLF3, ZNF741	Repressor	Ubiquitous	Cancers (breast, kidney, liver, ovary, prostate, stomach)	EMT
**KLF9**	BTEB, BTEB1	Activator	Ubiquitous	Demyelinating disorders, different types of cancers (brain, colon, intestine, multiple myeloma, uterus)	Thyroid hormone regulation
**KLF10**	TIEG, TIEG1, EGRα	Activator/Repressor	Ubiquitous	Angiogenesis, cardiac hypertrophy, different types of cancers (breast, kidney, pancreas, prostate)	TGFβ induced growth inhibition
**KLF11**	F-KLF, TIEG2, MODY7	Activator/Repressor	Ubiquitous	Liver fibrosis, type 2 diabetes, different types of cancers (leukemia, breast, colon, intestine, kidney, lung, ovary, pancreas, stomach)	TGFβ induced growth inhibition
**KLF12**	AP-2rep, AP2REP, HSPC122	Repressor	Brain, kidney, liver, lung	Head and neck cancer, stomach progression of gastric cancer, salivary gland tumors, autosomal dominant polycystic kidney disease (ADPKD)	Unknown
**KLF13**	BTEB3, NSLP1, RFLAT-1	Activator/Repressor	Ubiquitous	Head and neck Cancer	Unknown
**KLF14**	BTEB5, SP6, EPFN	Activator/Repressor	Ubiquitous	Type 2 diabetes	Transcription of TGFβRII
**KLF15**	K-KLF	Repressor	Ubiquitous	Glomerular disease, cardiovascular disease, kidney fibrosis	Cardiac fibrosis
**KLF16**	BTEB4, NSLP2, DRRF	Repressor	Ubiquitous	Adipose tissue expansion	Growth control mechanisms in NHK cells
**KLF17**	ZNF393	Repressor	Testis, brain, and bone	Cancers (metastasis in breast cancer, lung, hepatocellular carcinoma (HCC), gastric cancer, papillary thyroid carcinoma)	Downstream mediator of the TGF-β signaling pathway, anti-metastasis
**KLF18**	Unknown	Unknown	Unknown	Unknown	Unknown

Sp, specificity protein; Btd, buttonhead; KLF, Kruppel-like factor; EKLF, erythroid Kruppel-like factor; LKLF, lung [ED highlight—please note, capitalization varies, please choose one form and apply consistently.] KLF; OSX, osterix; BKLF, basic KLF; GKLF gut-enriched KLF; EZF, epithelial zinc finger; BTEB2, basic transcription element binding protein 2; IKLF, intestinal-enriched KLF; CPBP, core promoter-binding protein; Zf9, zinc finger 9; GBF, GC-rich sites binding factor; UKLF, ubiquitous KLF; BTEB, basic transcription element binding protein; TIEG1, Transforming growth factor beta -inducible early gene 1; EGRaTF, early growth response a transcription factor; TIEG2, TGF-β-inducible early gene 2; MODY7, maturity-onset diabetes of the young 7; NSLP, novel SP1-Like Protein; AP-2rep, AP-2 repressor; BTEB3 basic transcription element-binding protein-3; FKLF2, fetal-like globin gene-activating Kruppel-like factor-2; RFLAT-1, RANTES factor of late-activated T lymphocytes 1; KKLF kidney enriched KLF; BTEB4 basic transcription element-binding protein-4; DRRF dopamine receptor regulating factor; Zfp393, zinc finger protein 393.

**Table 2:** KLF10 role in diseases other than cancer.

Disease	TGFβ Signaling	Comments	Reference
Bone diseases	RANKL RUNX2 Smad2 ↓ Smad7 ↑	**Osteopenia:** KLF10plays a critical role in osteoblast-mediated mineralization and osteoblast support of osteoclast differentiation	[34]
	TGF-β1, BMP2, EGF	**Osteoblast:** KLF10 plays an active role in mediating Runx2 responses following TGFβ1 and BMP2.	[35]
Type 2 diabetes	KLF10, smad7 (weakly contributes)	KLF10 variants make minor contributions to a particular genetic background that increases susceptibility to the development of T2D.	[36]
Hypertrophy	Pttg1 ↑ (via Sp1 binding sites)	KLF10−/− mice develop a cardiac hypertrophic phenotype with asymmetric hypertrophy, interstitial fibrosis, and myocyte disarray.	[37]
Immune system	TGF-β1 and Foxp3 ↑	Loss of KLF10 enhanced CD4+ CD25 T cell activity, which stimulated inflammation and atherosclerosis and increased peripheral proinflammatory cytokines	[38]
Wound healing	Smad 7 ↑ Smad 2, 3 ↓	KLF10−/− mice delay wound healing. KLF10 may play a role in dermal wound healing via the TGFβ/Smad pathway.	[39]
NASH	TGFβ ↑ ChREBP ↓	Expression of KLF10 significantly increases in diet-induced NASH and ECM producing activated HSCs.	[40]
Colitis	KLF10, smad2, TGFβRII ↓	KLF10 regulates TGFβRII expression in murine macrophages via histone H3 modification.	[41]
Hyperglycemia	KLF10, Pgc-1α, Blood glucose ↑	KLF10 is an important regulator of hepatic glucose metabolism in mice.	[42]

↑ increase; ↓ decrease.

**Table 3:** KLF10 role in various cancer.

Cancer Type	Role	Comments	TGFβ Signaling	Reference
Prostate cancer	Suppressor	Doxazosin-mediated apoptosis in prostate cancer cells involves activation of KLF10 and Smad4 mRNA levels, as well as a decrease in Smad7 mRNA expression	Smad dependent pathway	[71]
Colorectal cancer	Suppressor	KLF10 is one of the members of the signal transduction of PPARγ pathway	Bcl2	[72]
Breast cancer	Suppressor	KLF10 plays an inhibitory role in the proliferation of breast cancer. KLF10 and Smad7 in breast cancers are inversely correlated	Smad7 ↑,KLF10, Smad2, and Bard1 ↓	[73]
Lymphoma cells	Suppressor	The participation of Smads in TGFβ induced apoptosis is supported by the increased expression of KLF10, which can activate the mitochondrial apoptotic pathway by increasing the intracellular level of ROS	KLF10 ↑Smad 2,3 ↓	[74]
Brain cancer	Suppressor	KLF10 is involved in apoptosis and was expressed at low levels in metastatic brain tumors.	Transactivator of TGFβ	[75]
Leukemia cells	Suppressor	KLF10 promotes apoptosis through the mitochondrial apoptotic pathway.	BimBax ↑Bcl2, Bcl-xl ↓	[31]
Renal cell carcinoma	Suppressor	KLF10 up-regulates the expression of TGFβI in von Hippel-Lindau gene (VHL) deficient tumors. KLF10 is a target of VHL	TGFβ1 ↑	[76]
Pancreatic cancer	Suppressor	Overexpression of KLF10 induced by the lentivirus system inhibited pancreatic cancer cell growth in vitro and in vivo.	G1-phase arrest in vitro	[77]
	Activator	Mutational screening of KLF10 in 22 pancreatic cancer cell lines revealed no alterations in expression.	No change in KLF10 expression	[78]
Hepatocellular carcinoma (HCC)	Suppressor	Upregulation of KLF10 in the HCC cell line induces inhibition of cellular proliferation	Smad3 Smad 7	[79]
	Activator	Deficiency of KLF10 suppresses cellular proliferation of hepatocytes during liver tumorigenesis through the TGF-β/Smad pathway	Smad 3 TGFβ1, TGFβ R1 ↑	[80]
Ovarian cancer	Suppressor	KLF10 displays strong BMAL1-dependent circadian expression; the KLF10 promoter recruits BMAL1 and is transactivated by the CLOCK/BMAL1 dimer through a conserved E-box response element.	An interruption in Circadian genes	[81]
Non–small cell lung carcinoma (NSCLC)	Suppressor	KLF10 suppresses TGFβ-induced EMT in conjunction with SNAI2 and HDAC1.	KLF10 ↓ SNAI1 ↑TGFβ/SMAD signaling ↑	[82]
Skin	Suppressor	Loss of KLF10 leads to enhanced tumor formation and progression.	P21 ↑ transcriptional activation in a p53 independent manner.	[83]
Multiple myelomas	Suppressor	MicroRNA-410 accumulation regulates cell proliferation and apoptosis by targeting KLF10 via activation of the PTEN/PI3K/AKT pathway in multiple myeloma.	PTEN/PI3K/AKT pathway	[42]

↑ increase; ↓ decrease.
